# Transcriptional responses of wheat roots inoculated with *Arthrobacter nitroguajacolicus* to salt stress

**DOI:** 10.1038/s41598-018-38398-2

**Published:** 2019-02-11

**Authors:** Maryam Safdarian, Hossein Askari, Vahid Shariati J., Ghorbanali Nematzadeh

**Affiliations:** 1 Department of Plant Molecular Physiology, Genetics and Agricultural Biotechnology Institute of Tabarestan, Sari Agricultural Sciences and Natural Resources University, Sari, Mazandaran Iran; 2 Department of plant sciences and biotechnology, Faculty of life Sciences and Biotechnology, Shahid.Beheshti University, G. C., Tehran, Iran; 3 Genome Center, National Institute of Genetic Engineering and Biotechnology, Tehran, Iran

## Abstract

It is commonly accepted that bacteria actively interact with plant host and have beneficial effects on growth and adaptation and grant tolerance to various biotic and abiotic stresses. However, the mechanisms of plant growth promoting bacteria to communicate and adapt to the plant environment are not well characterized. Among the examined bacteria isolates from different saline soils, *Arthrobacter nitroguajacolicus* was selected as the best plant growth-promoting bacteria under salt stress. To study the effect of bacteria on wheat tolerance to salinity stress, bread wheat seeds were inoculated with *A*. *nitroguajacolicus* and grown under salt stress condition. Comparative transcriptome analysis of inoculated and un-inoculated wheat roots under salt stress showed up-regulation of 152 genes whereas 5 genes were significantly down-regulated. Many genes from phenylpropanoid, flavonoid and terpenoid porphyrin and chlorophyll metabolism, stilbenoid, diarylheptanoid metabolism pathways were differentially expressed within inoculated roots under salt stress. Also, a considerable number of genes encoding secondary metabolites such as phenylpropanoids was detected. They are known to take part in lignin biosynthesis of the cell wall as well as antioxidants.

## Introduction

Crop yields and biomass is severely reduced by salt stress, primarily causes ionic imbalances leading to harmful effects on nutrient K^+^ attainment, water uptake, photosynthesis, enzyme activities and metabolism in the presence of high concentrations of Na^1^. Salt stress causes significant decreases in growth and productivity of crops in many areas. Hence, it is important to identify the mechanisms that grant tolerance to high salt environments^2^. In fact, salt is one of the main causes of abiotic stress, which limits agricultural production. In addition to creating plant ions imbalances and osmotic stress, excessive salinization prevents essential metabolisms including photosynthesis, protein and lipid synthesis, resulting in limited product and yield and even death of plant^3,4^.

Plant growth-promoting rhizobacteria (PGPRs) are soil bacteria which colonize plant roots and promote host growth either indirectly or directly through solubilization of phosphate and production of phytohormones under diverse environments. There are various PGPR-induced changes in plants growth promotion and adaptation as a result of complex combination of mechanisms affecting both plant development and nutrition^5,6^.

PGPRs induce changes in plants, and growth promotion due to a complex combination of various PGPR-induced mechanisms that affect both plant development as well as plant nutrition such as production of siderophores for iron absorption, plant hormones such as auxins and cytokinins, solubilizing phosphates, minerals and nutrients^7–9^. In addition, they facilitate plant growth under drought, heavy metals, flood and especially high salinity stresses by reducing the stress through the production of diminase-1-amino-cyclopropane-1-carboxylate (ACC) and altering the selectivity of K^+^, Na^+^ and Ca^2+^ and maintain a higher K^+^/Na^+^ ratio^10,11^.

In addition, in order to clarify molecular mechanism changes in plants related to PGPR mediated plant growth, transcriptomic analyses have been carried out for a small number of rhizobacterial species. To take further steps in understanding PGPR as an efficient tool for the agricultural field, primary mechanisms utilized by the stated bacteria should be examined extensively.

Promotion of tolerance to salinity occurs through various mechanisms, including the synthesis of compounds like osmolytes and polyamines, the attenuation of reactive oxygen species (ROSs) by antioxidants, the synthesis of polyamines transporting the ion homeostasis and compartmentalization^12–14^, nitric oxide formation^15^ and the synthesis and modulation of phytohormones^16,17^. Previous studies have shown PGPR-mediated salinity tolerance in host plant via the selectivity altering Na^+^, K^+^, Ca^2+^ amount and sustain a higher K^+^/Na^+^ ratio in plants. Many salt responsive genes are responsible for the mentioned mechanisms via physiological and biochemical variations; such as structural protein-coding genes like late embryogenesis abundant (LEA) proteins, osmoregulatory genes, antioxidant proteins, and transporters/antiporters such as high-affinity K+ transporter (HKT), Transcription factors (TFs) such as ERF, WRKY and signal-related protein kinases^18^ actint through some important pathways such as the salt overly sensitive (SOS) pathway^19,20^.

The purpose of this research was to investigate the gene expression patterns responsible for induced salt tolerance in wheat inoculated with PGPR compared to control using mRNA-seq. To the best of our knowledge, this is the first research to investigate the transcriptome to detect significantly differentially expressed genes and pathways in roots exposed to *Arhrobacter* sp. Unraveling of the primary mechanisms employed by the bacteria will hasten the recognition of PGPR as efficient and appropriate supplements to agricultural practice.

## Materials and Methods

### Isolation of rhizobacteria

Soil samples were collected from the rhizosphere of halophyte plants such as *Salicornia* spp, *Echinochloa*, *stagnina* and *Tamarix* L from the saline deserts of Iran, salinity range (23-110 dS/cm), and were suspended in sterile saline solution and shaken on an orbital shaker at 100 rpm for 20 min at room temperature. The suspensions of soil were diluted and 100 µl of each suspension was cultured on several medium plates such as yeast extract mannitol agar (YEMA), King’s B (KB) agar, nutrient agar (NA), water yeast extract agar (WYE), glycerol yeast extract agar (GYA), Luria bertani agar (LBA), triptic soy agar (TSA), eosin methylene blue (EMB). the plates were supplemented with 5% NaCl and incubated at 30 ± 2 °C for 3 days. A representative of each colony based on colony morphology (size, color, shape and growth pattern after 24 h of growth) was selected and transferred to liquid non-specific NA medium to establish pure cultures. The isolates were kept in 50% (v/v) glycerol at −80 °C.

### Plant Growth Promoting characterization

Isolated bacterial strains were tested for plant growth promoting characters. The phosphate solubilizing activity was tested on Pikovskaya (PVK) medium. IAA was measured according to the method developed by Brick *et al*.^20^ using Salkowsky’s reagent in tryptophan amended medium. ACC-deaminase activity test was performed using Dworkin and Foster (DF) minimal salts, according to the method developed by Penrose and Glick^21^ Accordingly, the best isolate demonstrating in *vivo* and in-*vitro* PGP ability was selected and characterized by *16SrDNA* gene partial sequence analysis. DNA was extracted by DNA extraction kit (Bioneer, South Korea) and PCR amplification was performed using universal Forward Primer 8 f (5′-AGAGTTTGATCCTGGCTCAG-3′) and 1492r (5′-GG(C/T)TACCTTGTTACGACTT-3′)^22,23^. PCR products were purified using PCR purification kit (Bioneer, South Korea) (ThermoFisher Scientific) and sequenced on an automated sanger sequencer by SeqLab Laboratory (Germany). sequence of the isolates was compared with the Gen Bank database of in the NCBI (http://www.ncbi.nlm.nih.gov) using BLASTn andPhylogenetic analysis was performed using MEGA 6 software (Molecular Evolutionary Genetic Analysis)^23^ by neighbour-joining method and 1,000 bootstraps.

### Plant inoculation and treatments

Wheat (*Triticum aestivum* L.) seeds were surface-sterilized (soaking in 70% ethanol for 2 min, followed by soaking in 1% sodium hypochlorite for 10 min), and rinsed four times with sterile distilled water and then planted in a 50 mL bottle. Bacterial strains were grown in NB medium at 30 °C and diluted to a final density of 10^8^ colony forming units (CFU) ml^−1^ in sterile distilled water containing 0.025% of Tween 20. Seeds were inoculated with the bacterial suspensions (10^8^ CFU ml^−1^) in a steril environment. Pots were filled with sterile clay soil and placed at temperature 31 °C and average humidity of less than 30% and divided to pots containing seeds with (1) bacteria inoculation and (2) without bacterial inoculation. The experiment was performed as a randomized complete block design with five replications for each group in greenhouse condition and The pots were watered daily for the first two weeks after germination with sterile water and then NaCl increased to NaCl at a concentration of 200 mM. seven days after germination, plants were irrigated with Hoagland nutrient solution supplemented with 0 and 200 mM NaCl for 7 days. The solution was applied in increments in order to not shock the plants with the added saline solution, maintaining soil water content at a constant value of about 70–75% of soil water holding capacity.14 days old seedlings were harvested and evaluated for parameters such as shoot dry biomass, root dry biomass and total dry. for all inoculated and un-inoculated plants under control and saline conditions. The concentration of Na and K (mg g^−1^) were determined in dry weight of leaves and roots after dry-ashing (550 °C) and digested in an acid mixture (HNO_3_:H_2_SO_4_:HClO_4_ = 10:1:3)^24^. Ethylene production was estimated following the protocol of Siddike *et al*.^25,26^.

### RNA extraction, library construction and sequencing

For each biological replicate, five root sections of roughly 5 cm in length from three locations on each plant root system were randomly collected and were pooled for RNA extraction. All sampled tissues were frozen in liquid nitrogen and stored at −80 °C. Total RNA of samples was extracted using Trizol Reagent (Invitrogen) following the manufacturer’s instructions.

RNAs purity (260/230 and 260/280 ratios) and integrity were assessed using NanoDrop Spectrophotometer (Termo Scientifc, MA) and an Agilent 2100 Bioanalyzer (Agilent Technologies, CA), respectively and was quantifed using Qubit® RNA HS Assay Kit by Qubit® 3.0 Fluorometer (Life Technologies, CA). High quality RNA of the inoculated and un-inoculated samples under saline condition were sent to Beijing Genomics Institute for mRNA-Seq using illumina TruSeq RNA Library Prep Kit and Illumina HiSeq2500 platform (Illumina, CA) as paired-end of 2 × 125 bps. The original sequencing datasets have been deposited in the European Nucleotide Archive (ENA) with the accession number SUB1231284.

### Sequence analysis

The quality of raw reads was inspected by FastQC (http://www.bioinformatics.babraham.ac.uk/projects/fastqc) and then subjected to Trimmomatic software version 0.32 to remove adapters, poly-N homopolymers and low-quality bases using a minimum read length of 50 bp an average quality cut-off of 30 (Phred score). Gene model annotation files and wheat reference genome was downloaded from ensemble website (ftp://ftp.ensemblgenomes.org/pub/plants/release-31/fasta/triticum_aestivum/dna/). Subsequently, Bowtie v2.2.3 was applied to construct an index of the reference genome and TopHat v2.0.12 to align the paired-end clean reads to the reference genome.

### Statistical analysis of differentially expressed genes (DEGs)

The cufflink v2.2.1 package was used to construct transcripts and count the reads mapped for the genes. Fragments per Kilobase of exon model per Million reads mapped (FPKM) were estimated for each gene and cuffldiff v2.2.0 package^27^ was applied for differential expression analysis. The false discovery rate was assessed using Benjamini and Hochberg multiple comparison correction. Genes with an adjusted P-value <0.05 and a fold change value ≥2 were considered as differentially expressed genes between the two groups.

### Gene Ontology and pathway enrichment analyses

The gene ontology (GO) terms of DEGs were retrieved using local databases and Gene Ontology Consortium (http://www.geneontology.org). The GOseq R package was used to analyze GO enrichment of DEGs as the gene length bias was corrected. Kyoto Encyclopedia of Genes and Genome (KEGG) pathways (KEGG, http://www.genome.jp/kegg/)^28^ were determined by in–house scripts of NIGEB Genome Center using David and KEGG databases.

### Quantitative real-time PCR

Expression of 9 up-regulated genes and two down regulated genes and representative of the metabolic pathways’ mechanism was investigated through Real-time RT-PCRs analyses (Supplementary Table S3). The cDNA of inoculated and un-inoculated wheat root samples was synthesized starting from 800 ng of total RNA using random nonamers. An iScript cDNA synthesis kit (Bio-Rad) produced the first-strand cDNA. To perform the quantitative real-time PCR, SsoFast EvaGreen Supermix Kit (Bio-Rad) on a CFX 96 Real-Time System (Bio-Rad) was used for the samples. Each 10 μl PCR reaction contained 0.6 μl of each primer (10 μM), 1 μl of diluted cDNA, 1X SYBR Green Real-Time PCR Master Mix (Thermo Fisher Scientific) and sterile water. Thermal cycling conditions were: 95 °C for 10 min followed by 40 cycles of 94 °C for 15 s, 58 °C for 30 s, and 72 °C for 30 s, and a melt cycle with 1 °C increments from 55 to 96 °C. The expression of each gene in different treatments was calculated by comparing their 2^−ΔΔCt^ values.

## Result

### Isolation of rhizobacteria

**Figure 1:**
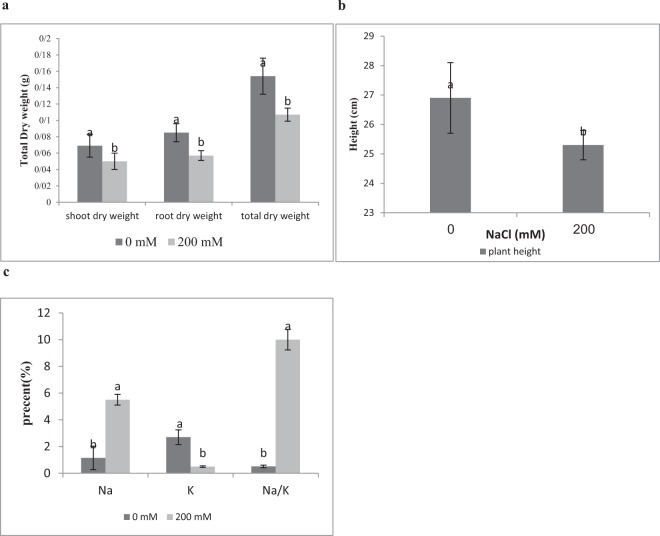
(**a**,**b**) shoot dry weight, root dry weight total dry weight and height of plants grown in 0 and 200 mM NaCl after germination, (**c**) The Na^+^, K^+^ and Na^+^/K^+^ concentration in the shoots of plants treated with 0 and 200 mM NaCl. Different letters indicate statistically significant differences between treatments (Duncan’s multiple range tests, *P*  <  0.05).

**Figure 2:**
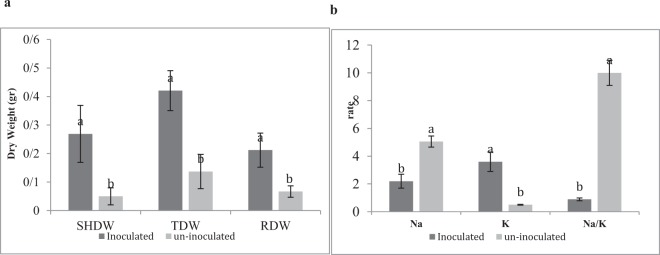
Effect of *A*. *nitroguajacolicus* inoculation on wheat growth traits: (**a**) SHDW (shoot dry weight), TDW (total dry weight) RDW (root dry weight), *A*. *nitroguajacolicus* increased dry wheight (**b**) Na+, K+ and Na+/K+ concentration in shoot plants treated 200 mM NaCl. *A*. *nitroguajacolicus* decreased Na+ and increased K+ concentration. Different letters indicate statistically significant differences between treatments (Duncan’s multiple range tests, *P*  <  0.05).

**Figure 3:**
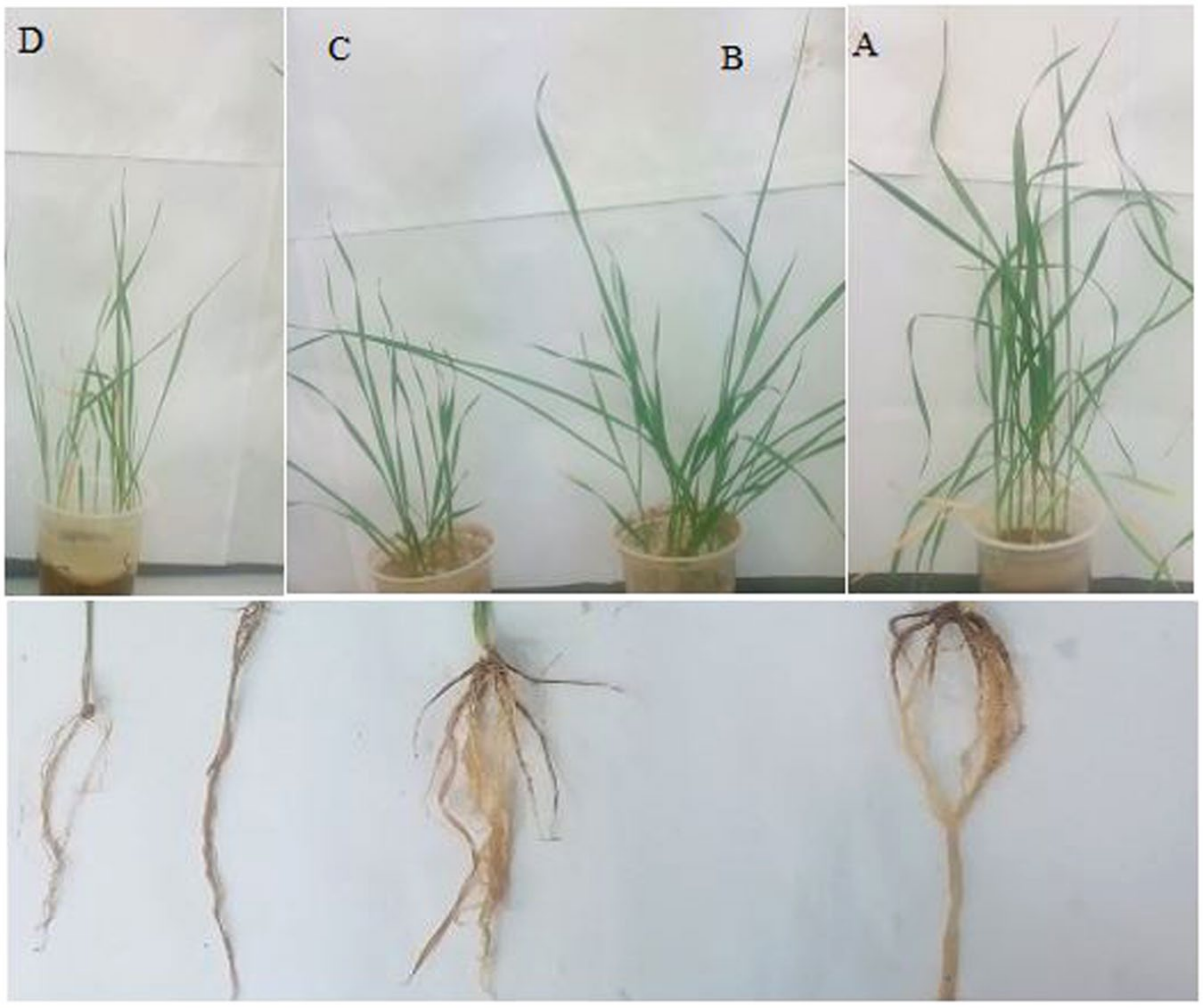
Phenotypical comparisons of inoculated and un-inoculated plants in response to salt treatment. The phenotypes (**A**) shoot and root of *A*. *nitroguajacolicus* inoculated plants under saline condition (200 mM NaCl) (**B**) shoot and root of *A*. *nitroguajacolicus* inoculated plants (**C**) shoot and root of control plants (**D**) shoot and root control plants under saline condition (200 mM NaCl).

In initial screening process, 30 isolates were investigated for the ability of phosphate solubilization, IAA, siderophore, HCN production, ACC deaminase activity and increased wheat dry weight under salinity stress (Supplementary Tables S1). Wheat plants treated with bacterial isolate 83 produced maximum shoot dry weight and total dry weight under salt stress. Plant growth promoting traits like phosphate solubilization, indole acetic acid (IAA) production, siderophore production and ACC deaminase activity were shown in Table 1. A decrease in shoot dry weight, root dry weight, total dry weight, plant height, Na^+^ content and Na^+^/K^+^ rate was observed in plants under salt stress (200 mM NaCl) in comparison to control samples (Fig. 1). Isolate 83 treated plants total dry weight (261%), shoot (390%) and root dry weight (270%) increased comparing to control treatment while levels of ethylene (24%) decreased in salinity condition (Figs 2,3, Table 2). Inoculation with isolate 83 reduced Na^+^ and increased K^+^ in the shoots significantly as compared to the control (Fig. 2).

**Table 1:** Plant growth promoting characters of *Arhrobacter nitroguajacolicus*. Values are means of three replicates ± standard error.

Isolate	Phosphate solubility (µg/ml)	pH	Sidrophore %	Auxin (µg/ml)	Acc deaminase (µmol α ketobutyrate mg protein-1 h-1)
*Arhrobacter nitroguajacolicus*	1797 ± 5.6	3.6 ± 0.2	38.0	20.7 ± 0.34	25.22 ± 0.3

**Table 2:** Ethylene biosynthesis by 14 days old wheat (*Triticum aestivum* L.) plantlets under different salt stress conditions, with and without inoculation of *Arhrobacter* sp. Average _ standard error from 3 replications. Different letters indicate significantly different means (LSD P < 0.05) within incubation times.

Treatment	Ethylene emission(nmol ethylene g FW^−1^ h^−1^)			
	4 h incubation		24 h incubation	
Nacl concentration	0 mM	200 mM	0 mM	200 mM
Un- inoculated	0.0 ± 0.0	73.23 ± 4.62	8.03 ± 2.20	11.56 ± 2.16
*Arhrobacter nitroguajacolicus*	0.0 ± 0.0	62.13 ± 6.60	7.33 ± 1.80	7.45 ± 0.74
LSD (0.05%)	0	12.3	1.6	2.3

### Monitoring ethylene emission of plantlets

Ethylene production significantly decreased in plants inoculated with isolate 83 at 200 mM NaCl comparing to un-inoculated ones. Maximum ethylene emission after 24 h incubation was detected in un-inoculated plantlets treated with 200 mM NaCl, compared to un-inoculated plantlets without NaCl (Table 2). The reduction of ethylene emission due to the inoculation with salt tolerant PGPR was statistically significant, and plantlets inoculated with isolate 83 showed a decrease of 35.35% in ethylene emission in 200 mM NaCl level at both 4 and 24 h after incubation (Table 2).

### Molecular characterization and phylogenic analysis of bacterial isolate

**Figure 4:**
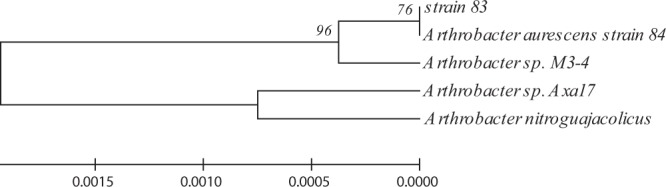
Phylogenetic analysis based on *16S rDNA* sequence available from EzTaxon-e database was constructed. Distances and clustering with the neighbor-joining method was performed by using the software MEGA version 6.0. Bootstrap values based on 1,000 replications (bootstrap values of <50 %, not shown) are listed as percentages at the branching points.

*16SrDNA* partial sequence analysis identified the isolate 83 as a member of the *Arhrobacter* genus, showing the best homology with *Arthrobacter nitroguajacolicus* with 99% similarity (Fig. 4).

### Transcriptome analysis overview

To unravel the mechanisms that *Arthrobacter nitroguajacolicus* induced salt tolerance in wheat plants, transcriptome profiling was performed using RNA-Seq approach, resulting in over 80 M raw reads from two inoculated and un-inoculated samples under salt stress (Supplementary Tables S2). Expression analysis between two treatments, identified 152 DEG genes (P-Value < 0.05); showing 147 genes up-regulated in inoculated plants and 5 genes were down-regulated. The highest up-regulated genes are AA1763630, AA0467650, calcium ion binding, Nicotianamine synthase, AA0122100, AA2042560, AA0607960, zinc ion binding and Cytochrome P450.

Eight up-regulated genes (Supplementary S4) involved in Nicotianamine synthase were known to be involved in Iron (Fe) acquisition. Phosphatase genes (AA0975030, AA1182960) that is induced by phosphate starvation and oxidative stress conditions were up-regulated in inoculated plants (Supplementary S4).

Moreover, several transporters involved in the plant salt stress response such as ion transporters (calcium, zin, ctr copper and sulphate), sugar transporter, ABC transporter, oligopeptide, Amino acid/polyamine and Folate-biopterin transporter were up-regulated (Supplementary S4). Main down-regulated genes were RlpA-like protein, Cytochrome P450 (2) and metallothionein beloning to cysteine and methionine metabolism was, glycerolipid metabolism, biosynthesis of secondary metabolites and metabolic pathways (Supplementary S5).

**Figure 5:**
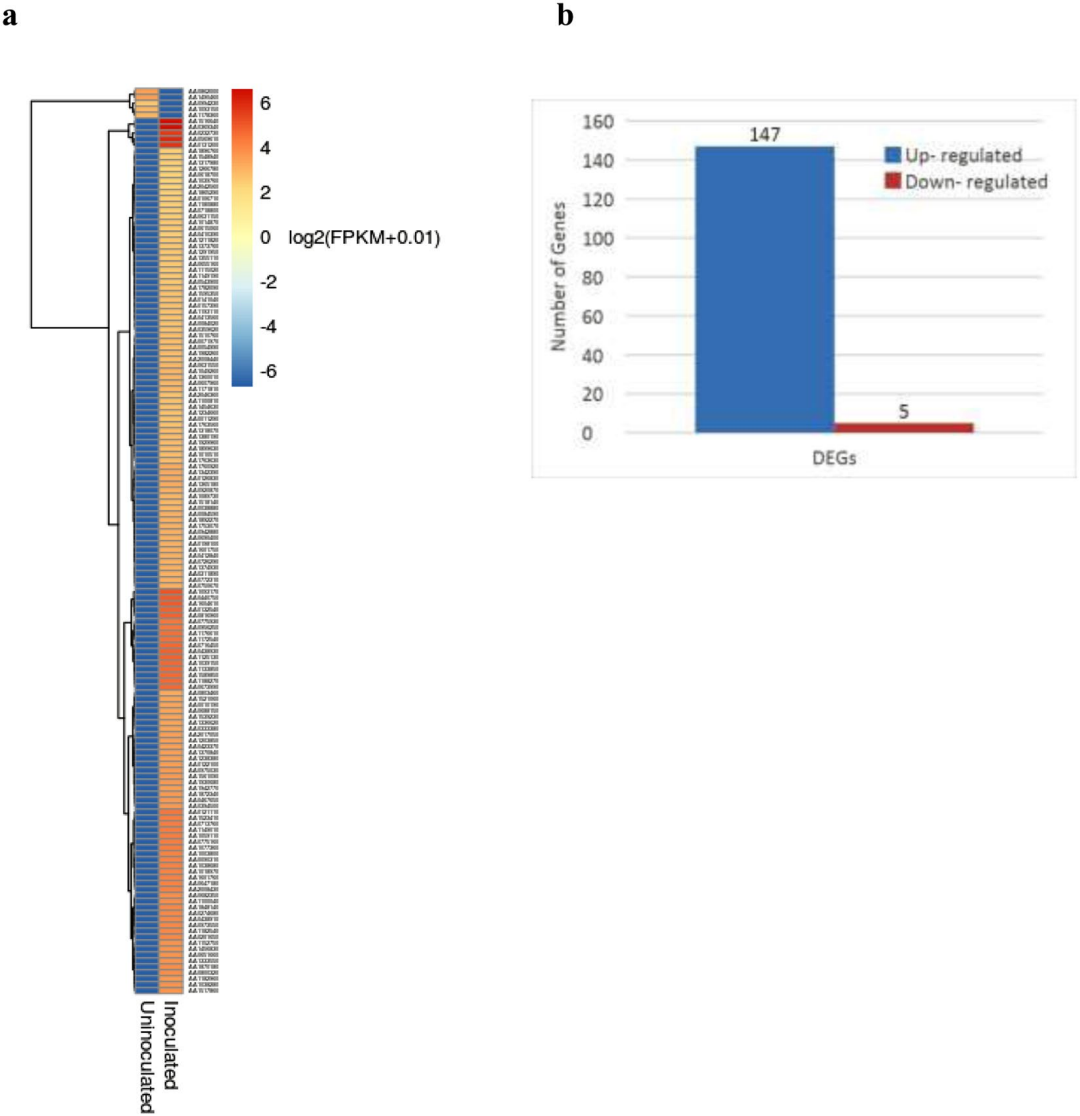
(**a**) Heat-maps of the log2 Ratio values of salt responsive DEGs were used for hierarchical cluster analysis with heatmap. Gene expression values are scaled ranging from -6 (blue) to 6(red). red represents up-regulated gene, blue represents down-regulated genes. (**b**) Numbers of downregulated and upregulated differentially expressed (DE) genes after salt stress induction from comparisons of control and inoculated samples. Comparison of salt-responsive gene id co-expressed in NaCl-stressed inoculated and un-inoculated roots from the two clusters using hierarchical cluster analysis.

GO annotation and statistical analyses demonstrated that 152 significant DEGs were classified into three GO ontology’s classes and 121 terms (Fig. 5 and Supplementary S6). the most significant (corrected p-value < 0.005) enrichment terms of up-regulated genes in the biological process category were metabolic process (GO:0008152), biological regulation (GO:0065007), biosynthetic process (GO:0009058), cellular aromatic compound metabolic process (GO:0006725), cellular nitrogen compound metabolic process (GO:0071495), gene expression (GO:0010467), heterocycle metabolic process (GO:0046483), macromolecule metabolic process (GO:0043170), nucleic acid metabolic process (GO:0090304), organic cyclic compound metabolic process (GO:1901360), proteolysis (GO:0006508) and regulation of metabolic process (GO:0019222).

**Figure 6:**
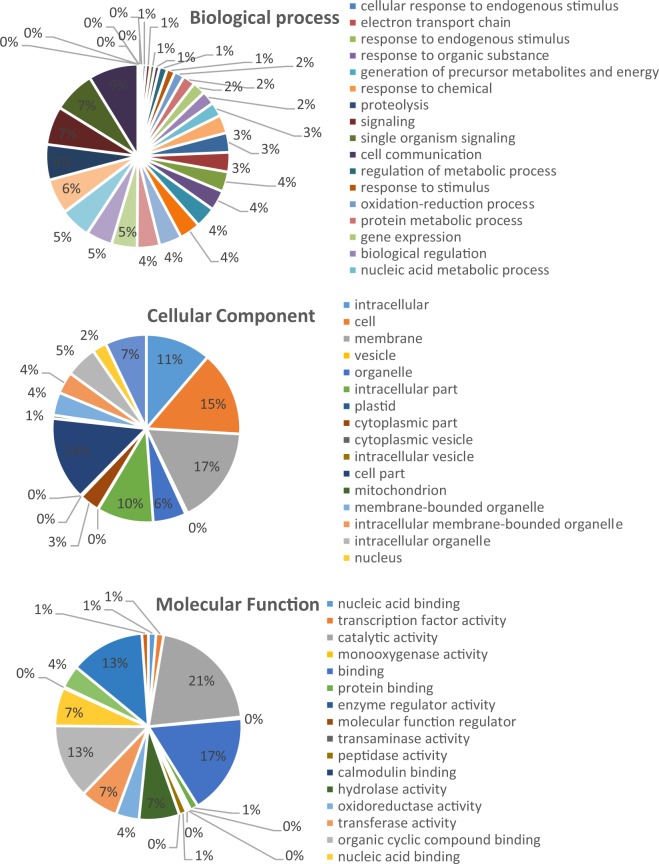
Pie charts showing the Gene Ontology (GO) analyses of commonly differentially expressed genes. The differentially expressed genes were assigned into three groups, including biological process, cellular components and molecular function.

Other significantly (corrected p-value < 0.05) enriched categories included cellular macromolecule metabolic process (GO:0044260), nitrogen compound metabolic process (GO:0006807), organic substance biosynthetic process (GO:1901576), organic substance metabolic process (GO:0071704) and primary metabolic process (GO:0044238). GO enrichment analysis in molecular function category showed for catalysis enzymes as the most significant class that includes up-regulated genes such as those for aspartic-type endopeptidase activity, aspartic-type peptidase activity, transferase activity and transferring nitrogenous groups (Fig. 6 and Supplementary S6).

### Metabolic Pathway Analysis

**Figure 7:**
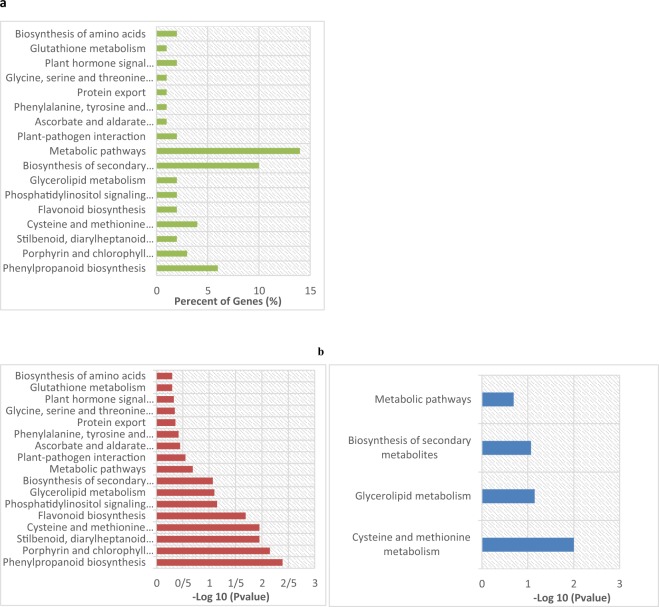
(**a**) GO classification of *T*. *aestivum* transcriptome and differentially expressed genes between control and inoculated root in salt stress. The Y-axis represents number of unigenes and X-axis shows the GO categories. (**b**) Pathway assignment based on the Kyoto Encyclopedia of Genes and Genomes (KEGG) database.

**Figure 8:**
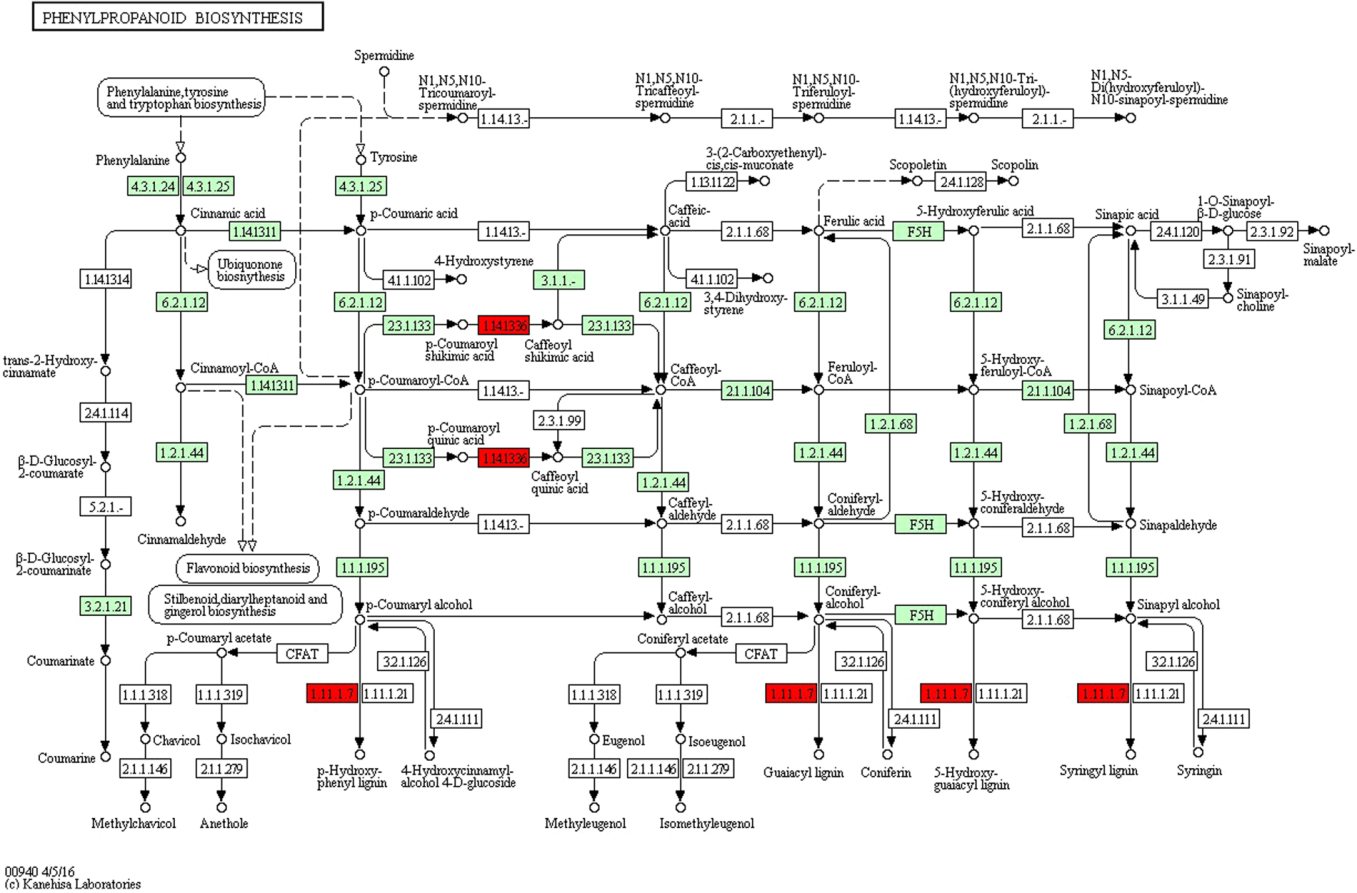
Examples of KEGG pathway of phenylpropanoid found for transcripts associated with metabolic pathways. Each box shows enzymes involved in each section of the pathway. Genes highlighted in red were differential experssion.

Assignment of significant DEGs to KEGG pathways showed the most significant pathways were phenylpropanoid biosynthesis (Fig. 7 and Supplementary S5) which leads to the biosynthesis of lignin. In our data, a number of genes for lignin biosynthesis included four peroxidases (AA0410390; AA1982260; AA0412840 and AA1872340) are involved in plant cell wall biosynthesis and two cytochrome P450 (AA0618700; AA0359620) (Fig. 8 and Supplementary S5) were upregulated in inoculated plants.

Five down regulated genes in inoculated roots were assigned to cysteine and methionine metabolism, biosynthesis of secondary metabolites, glycerolipid metabolism and metabolic pathways (Fig. 5 and Supplementary S5).

### Validation of RNA-Seq results by qRT-PCR

**Figure 9:**
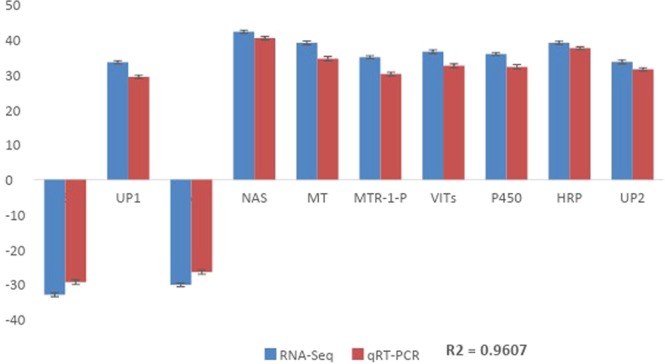
Validation of RNA-Seq results using qRT-PCR. The name of transcript factor behind each gene is determined by different expression gene function annotation.

Differential expression of 11 candidate genes based on significant DEG between inoculated and un-inoculated groups was validated through Real-time RT-PCR (Fig. 9). Nine genes showed up-regulation (fold change ranged between 29.3 and 40.4) and two genes were down-regulated (fold change ranged between −26.4 and −29.3). A highly significant correlation was found between the RNA-seq and real-time RT-PCR results (P = 0.001, R2 = 0.9607). These results suggest that the investigated genes are indeed related to the response of the inoculated plants to induced salt tolerance.

## Discussion

Based on reported studies, rhizobacteria can play a significant role in increasing plant growth and reducing different abiotic stresses effects such as drought and salinity. Bacterial species that are resistant to salinity and high temperatures can lead to an increase in yields and plant’s production^29,30^. *Arhrobacter* species promote plants growth by enhancing 1-aminocyclopropane-1-carboxylic acid (ACC), indole acetic acid (IAA) production and siderophore and the ability to solubilize phosphate (Ca_3_(PO_4_)_2_). Furthermore, some observed features such as the ability to form biofilm and utilize various components of plant root exudates like sugars, amino acids and organic acids clearly represent its lifestyle as a plant rhizosphere associated bacterium^31^. In this study a potential promoting synergistic interaction with rhizobia was observed for *A*. *nitroguajacolicus*, resulting in a greater ratio of dry weight of wheat plant tissues under salt stress (Fig. 2).

The present study aimed to identify the key genes and pathways contributing in mediated salt tolerance of wheat plants through halotolerant rhizobacteria *Arthrobacter nitroguajacolicus*. Based on the results, the bacteria could promote the plant growth by improving the dry weight and root length under both non-saline and saline conditions. Furthermore, the plants colonized by bacteria could enhance the tolerance of salinity (Supplementary Table S1).

Transcriptome wide analysis of plants influenced by *Arhrobacter* sp in saline stress showed upregulation of genes largely involved in cell, cell part and metabolic process, leading to a stress response in plant. The phenylpropanoid pathway was one of the most enriched pathways with a large number of differentially expressed genes in inoculated root (Fig. 8 and Supplementary S5). A recent study has shown that colonization with *Piriformospora indica* increases activity of genes within the phenylpropanoid pathway^32^, which is responsible in lignin biosynthesis of the cell wall, antioxidant activity, and interactions with biotic and abiotic environments^33,34^. Cytochrome P450s are regarded as hemethiolate enzymes participating in the redox reaction and are included in a large number of biosynthetic pathways^35^. Our data showed P450s genes (CYP98A1, CYP734A5, CYP72A15 and CYP710A1) were up-regulated in inoculated samples under salt stress. CYP450 proteins are served as the signals for growth and development and are responsible for protecting plants from different biotic and abiotic stresses. Cytochrome P450 monooxygenases, which belongs to the CYP98 family, play a major role in catalyzing the meta-hydroxylation step in the phenylpropanoid biosynthetic pathway^36^. In addition, the repression in such gene may result in plant defense weakness and creating some phenolic compounds that modify lignin composition and impair plant development^37^.

Salt stress can play a significant role in stimulating an oxidative burst in plants as a primary immune response. An increase in reactive oxygen species (ROS) can be used as an alarm signal to start acclimation and defense reactions, which is maintained in a tight balance by the antioxidant systems in plants^38^. However, the oxidative burst may result in creating an extensive cellular damage and accordingly cell death if the salt stress lasts for a certain period of time^39^. In the present study, the expression of genes encoding ascorbate peroxidase (APX) was up-regulated significantly by bacteria inoculation under salt conditions (Fig. 8). The glutathione-ascorbate cycle is regarded as a metabolic pathway which plays a pivotal role in detoxifying hydrogen peroxide (H2O2) in plant chloroplasts^40^. APX and Glutathione peroxidase (GPX) detoxify hydrogen peroxide by using ascorbate and glutathione as substrates, respectively. Regarding the significant up-regulation of genes encoding APX and GPX synthesis, bacteria may protect plant chloroplasts through enhancing ROS scavenging capability. The increased levels of grass ROS scavenging enzyme activity by bacteria inoculation under saline stress have been reported in several bacteria-plant associations under salt stress. *Dietzia natronolimnaea* infection could increase superoxide dismutase (SOD), catalase (CAT), peroxidase (POD) and APX activities, and decrease H2O2 levels in *Elymus dahuricus* under water deficit^41^. Similarly, Previous works, concluded that wheat (*Triticum aestivum* L.) infected by *Bacillus amyloliquefaciens SQR9* can improve peroxidase/catalase activity and glutathione content and reduce Na+ levels in plants subjected to saline stress^42^. However, no study could prove whether the increased antioxidant enzyme activity is related to plant genes encoding these enzymes. However, as shown in present study, several APX and GPX genes in the glutathione-ascorbate cycle were up-regulated under saline stress (Supplementary S4). In other words, the regulation of antioxidant enzyme activity by the bacterial inoculation can play a significant role in enhancing the bacteria-mediated salt tolerance of plant.

Under salt stress, plants can maintain stable concentrations that permit cell metabolism by selective absorption, efflux, and regional integration of ions. Nicotianamine synthase gene (NAS) may play a major role in metal hyper-accumulation and hyper-tolerance among higher plants^43–47^. The expression of NAS is induced by salt stress and the gene role has been proven in iron absorption in plants^43^. NAS expression can be used to catalyze and synthesize niacinamide (NA), the synthetic precursor of plant iron carrier.

Maintaining homeostasis in the cytoplasm, which is usually related to reorganization and spatial distribution of a large number of key metabolites, is important for appropriate metabolic responses of plants under salt stress. In addition, rapid synthesis of osmolytes and efficient transport machinery should be taken into account for conducting the process^48^. Furthermore, the results of transcriptome analysis indicated that almost 10% of DEGs in wheat root encoding transporter proteins such as Oligopeptide transporters (OPTs), ATP binding cassette (ABC) transporters, Sugar/inositol transporter, ATPase, ion transporter, and aquaporin were up-regulated in inoculated plantlets under 200 mM NaCl stress. Oligopeptide transporters (OPTs) are regarded as membrane-localized proteins with transport capability of a wide range of substrates such as glutathione^49^ and metals^50,51^. In the present study, three genes responsible for encoding oligopeptide transporters (AA0010190, AA0090310 and AA0198100) were up-regulated (Supplementary S4). The ABC transporter superfamily is regarded as a class of ubiquitously distributed proteins which plays a significant role in mediating the energy-driven transport of a number of substances across the membranes. ABC transporter genes in *Arabidopsis* represent different responses at transcriptional level after conducting abiotic and biotic treatments^52^. The higher expression levels of ABC transporter genes under salt stress indicated that bacterial inoculation could enhance the transporting ability with respect to salt stress. In addition, controlling ionic homeostasis is considered as an important mechanism for salinity tolerance. Based on the results, Na+ influx transporter (HKT) and the tonoplast Na^+^/H^+^ antiporter (NHX) are involved in Na+ homeostasis and vacuolar compartmentation under salt stress in plants. HKT and NHX antiporter were up-regulated under salt stress in plants inoculated with PGPR such as *Dietzia natronolimnaea*^41^, *Bacillus amyloliquefaciens* SQR9^42^, *Serratia* sp. Sl-12^53^ and *Bacillus subtilis* GB03^54^. In order to understand spatiotemporal regulation of short and long-term salt stress, the results of another study conducted with *Arabidopsis thaliana* and *Burkholderia phytofirmans* PsJN indicated that colonized plants could demonstrate higher tolerance to the sustained salt stress. The expression patterns of genes involved in ion homeostasis (*KT1*, *HKT1*, *NHX2*, and *SOS1*) after the stress and rapid molecular changes induced by PsJN may be related to the observed salt tolerance^55^. Probably expression level of such antiporter including HKT and NHX seems efficient induced mechanisms for coping with salt stress by bacteria in wheat plantlet.

WRKY TFs play a crucial role in controlling a large number of stress induced reactions in plants^56,57^. However, discovering their roles in different abiotic stress responses has lagged behind, compared to biotic stresses. In the present study, PGPR-inoculated plants had higher WRKY28 genes expression in saline condition in comparison to un-inoculated plants, which may be regarded as a reason for providing tolerance effect under salt stress. WRKY TFs can play a significant role in regulating water/drought-stress by modulating the cellular osmotic balance, scavenging ROS mechanism, and expressing different stress-related genes as it was indicated by the enhanced and improved growth in PGPR applied plants under greenhouse conditions.

## Conclusion

Our results demonstrated that symbiosis of wheat with *A*. *nitroguajacolicus* increased shoot and root mass. Also, nutrient acquisition of key macronutrients, including N, P, and K, as well as of several micro and beneficial nutrients, especially Fe increased in inoculated plants. In this study, the bacteria also decreased Na absorption and ethylene level through increasing ACC deaminase level in wheat. Our study shows the improvement of salt stress in wheat by a plant growth promoting bacterium (*A*. *nitroguajacolicus)* which modulate expression of genes mainly involved in phenylpropanoid biosynthesis, porphyrin and chlorophyll metabolism, stilbenoid, diarylheptanoid and gingerol biosynthesis, cysteine and methionine metabolism, flavonoid biosynthesis, phosphatidylinositol signaling system, glycerolipid metabolism and biosynthesis of secondary metabolites pathways. Transcriptome analyses revealed that the presence of the *Arhrobacter sp* enhanced wheat plantlets salt tolerance by up-regulating expression of genes such as Cytochrome P450s, ascorbate peroxidase (APX), NAS, Oligopeptide transporters (OPTs), ATP binding cassette (ABC) transporters Sugar/inositol transporter, ATPase, ion transporter and etc.Those genes are crucial in biosynthesis of the cell wall and antioxidant activity, and play important roles in interaction and adaptation to biotic and abiotic environments. This work highlights the importance of application of halotolerant bacteria species isolated from saline soil to induce tolerance to salt stress and provides clue into mechanisms by which bacteria improve salt tolerance.

## Supplementary information

Supplementary files

Supplementary 6

Supplementary 4

Supplementary 5
