# Supplementary material for: Transcriptional responses of wheat roots inoculated with Arthrobacter nitroguajacolicus to salt stress
Source: Sci Rep. 2019 Feb 11;9:1792. doi: 10.1038/s41598-018-38398-2 (PMC6370872; doi:10.1038/s41598-018-38398-2)
Supplement: Supplementary file 1 — Supplementary files [file 41598_2018_38398_MOESM1_ESM.docx]

**Transcriptional responses of wheat roots inoculated with *Arthrobacter nitroguajacolicus* to salt stress**

Maryam Safdarian^a,c^, Hossein Askari^b^, Vahid Shariati J^c^, Ghorbanali Nematzadeh^a^

^a,^ Department of Plant Molecular Physiology, Genetics and Agricultural Biotechnology Institute of Tabarestan, Sari Agricultural Sciences and Natural Resources University,Sari, Mazandaran, Iran.

^b*^ Department of Biotechnology, Faculty of New Technologies and Energy Engineering, Shahid.Beheshti University, G. C., Tehran, Iran.

^c^ Genome Center, National Institute of Genetic Engineering and Biotechnology, Tehran, Iran

| **Supplementary Table S1.** Plant growth-promoting traits of salt-tolerant bacterial isolates. Values are means of three replicates ± standard error. | | | | | | |
| --- | --- | --- | --- | --- | --- | --- |
| **Isolate number** | **Phosphate solubility (µg/ml)** | **Auxin (µg/ml)** | **Sidrophore %** | **Acc deaminase (µmol α-ketobutyrate mg protein‒1 h‒1)** | **Total dry weight**  **(g)** | |
|  |  |  |  |  | **0 (mM)** | **200 (mM)** |
| 1 | 0.0 ±0.0 | 10.4 ± 0.18 | 0.0 ± 0.0 | 4.34 ± 0.12 | 0.154± 0.018 | 0.167± 0.018 |
| 2 | 0.0 ± 0.0 | 20 ± 0.18 | 0.0 ± 0.0 | 17.96 ± 0.9 | 0.386± 0.039 | 0.322± 0.028 |
| 3 | 147 ± 1.2 | 11.1 ± 0.18 | 0.0 ± 0.0 | 13.32 ± 0.4 | 0.35± 0.033 | 0.293± 0.029 |
| 7 | 1480 ± 8.6 | 25 ± 0.65 | 0.0 ± 0.0 | 19.82± 0.9 | 0.31± 0.047 | 0.288± 0.029 |
| 8 | 146 ± 0.9 | 25 ± 0.22 | 5 ± 0.58 | 1.89 ± 0.35 | 0.298± 0.031 | 0.236± 0.022 |
| 9 | 0.0 ± 0.0 | 10 ± 0.18 | 7 ± 0.59 | 23.23 ± 0.8 | 0.369± 0.031 | 0.295± 0.022 |
| 12 | 0.0 ± 0.0 | 18 ± 0.23 | 0.0 ± 0.0 | 12.89 ± 0.3 | 0.339± 0.048 | 0.24± 0.029 |
| 14 | 0.0 ± 0.0 | 19 ± 0.65 | 11 ± 0.71 | 9.22± 0.32 | 0.368± 0.035 | 0.323± 0.029 |
| 15 | 0.0 ±0.0 | 18 ± 0.2 | 14 ± 0.75 | 7.62 ± 0.2 | 0.394± 0.037 | 0.322± 0.033 |
| 19 | 0.0 ± 0.0 | 8 ± 0.07 | 0.0 ± 0.0 | 11.48± 0.3 | 0.387± 0.046 | 0.383± 0.029 |
| 20 | 0.0 ± 0.0 | 15 ± 0.23 | 0.0 ± 0.0 | 5.22 ± 0.5 | 0.325 ± 0.034 | 0.409 ± 0.052 |
| 21 | 0.0 ± 0.0 | 12 ± 0.18 | 0.0 ± 0.0 | 17.09 ± 0.3 | 0.347± 0.032 | 0.267± 0.025 |
| 23 | 0.0 ±0.0 | 20 ± 0.3 | 0.0 ± 0.0 | 21.22 ± 0.9 | 0.359± 0.032 | 0.331± 0.038 |
| 24 | 0.0 ± 0.0 | 12 ± 0.25 | 0.0 ± 0.0 | 12.29 ± 0.1 | 0.319± 0.027 | 0.257± 0.019 |
| 32 | 73.0±3.2 | 6 ± 0.04 | 0.0 ± 0.0 | 11.09 ± 0.1 | 0.637 ± 0.073 | 0.317 ± 0.031 |
| 38 | 0.0 ± 0.0 | 10 ± 0.17 | 0.0 ± 0.0 | 19.99 ± 0.6 | 0.414 ± 0.039 | 0.378 ± 0.041 |
| 39 | 0.0 ± 0.0 | 8.3 ± 0.06 | 0.0 ± 0.0 | 4.89 ± 0.7 | 0.416 ± 0.039 | 0.379 ± 0.043 |
| 40 | 1850 ± 5.3 | 8.2 ± 0.09 | 0.0 ± 0.0 | 3.34 ± 0.1 | 0.347 ± 0.045 | 0.248 ± 0.031 |
| 41 | 1202 ± 3.3 | 13.4 ± 0.22 | 0.0 ± 0.0 | 5.52 ± 0.7 | 0.343 ± 0.039 | 0.259± 0.032 |
| 42 | 0.0 ± 0.0 | 30 ± 1.2 | 0.0 ± 0.0 | 3.52 ± 0.1 | 0.362 ± 0.046 | 0.294± 0.031 |
| 47 | 1008 ± 6.2 | 4.1 ± 0.03 | 0.0 ± 0.0 | 22.89± 0.5 | 0.301± 0.026 | 0.323± 0.023 |
| 48 | 2012 ± 6.8 | 4.2 ± 0.06 | 6 ± 0.37 | 10.89 ± 0.4 | 0.416± 0.032 | 0.221± 0.029 |
| 53 | 1399 ± 5.4 | 48 ± 0.53 | 0.0 ± 0.0 | 12.3 ± 0.1 | 0.398± 0.045 | 0.336± 0.043 |
| 57 | 1667 ± 3.6 | 10 ± 0.18 | 0.0 ± 0.0 | 25.22± 0.3 | 0.462± 0.053 | 0.495± 0.058 |
| 65 | 1480 ± 4.6 | 18 ± 0.44 | 4 ± 0.25 | 18.85± 0.3 | 0.336± 0.032 | 0.276± 0.029 |
| 69 | 1377 ± 8.8 | 6.5 ± 0.05 | 0.0 ± 0.0 | 6.33± 0.61 | 0.312± 0.041 | 0.32± 0.029 |
| 76 | 388 ± 2.9 | 20.7 ± 0.34 | 0.0 ± 0.0 | 2.33 ± 0.52 | 0.41± 0.063 | 0.332± 0.044 |
| 83 | 1797 ± 5.6 | 121.7 ± 2.3 | 17 ± 0.85 | 34.28± 0.41 | 0.45± 0.034 | 0.421± 0.027 |
| 84 | 219 ± 1.4 | 78.3 ± 2.00 | 0.0 ± 0.0 | 2.66 ± 0.2 | 0.386± 0.045 | 0.407± 0.075 |
| 96 | 188 ± 3.1 | 8.3 ± 0.06 | 0.0 ± 0.0 | 33.25± 0.8 | 0.371 ± 0.036 | 0.267 ± 0.029 |
| LSD (0.05%) | 12.5 | 5.7 | 2.5 | 6.3 | 0.3 |  |

**Supplementary Table S2.** Summary of sequencing data.

| **Samples** | **Total Reads** | **Total Nocleotides** | **Q20 Precentages** | **N Precentage** | **GC Precentage** |
| --- | --- | --- | --- | --- | --- |
| un-inoculated1 | 20372157 | 40744314 | 94.04% | 0.0 | 54 |
| un-inoculated2 | 20372157 | 40884314 | 93.75% | 0.0 | 54 |
| Inoculated1 | 21593216 | 43297527 | 94.95% | 0.0 | 56 |
| Inoculated2 | 21704311 | 43297527 | 94.04% | 0.0 | 56 |

| **Supplementary Table S3**. DNA primers used for quantitative reverse transcription polymerase chain reaction. (qRT-PCR) analysis | |
| --- | --- |
| Genes | Primers (5’-3’) |
| TRIAE_CS42_4BS_TGACv1_328754_AA1093150 | F: CCACCTTGCAGTCTCTGACA  R: GGGGATGAAGTCGTTGAGAA |
| TRIAE_CS42_6AS_TGACv1_486422_AA1561090 | F: GACATCCACACCACGAAATG  R: GAAGGTGCTCCAGATGAAGG |
| TRIAE_CS42_3DL_TGACv1_250086_AA0862000 | F: AGCTTGTTTTGGCTGCGTAT  R: CCACTTGGTGATCTGCACTG |
| TRIAE_CS42_4DL_TGACv1_344693_AA1149010 | F: TCAAGCAAGAAGCTCCATCA  R: CGTGACCAGCTCAGTGAAGA |
| TRIAE_CS42_1BL_TGACv1_032546_AA0131200 | F: CCCTGACTTGGAGGAGAAGA  R: ATCACACACGGCACAATCAC |
| TRIAE_CS42_4BS_TGACv1_328755_AA1093170 | F: AAAGGCCTTCTGCACTGAAA  R: TTGTTGGCAGTGTCACCATT |
| TRIAE_CS42_1AL_TGACv1_000363_AA0010190  TRIAE_CS42_2AS_TGACv1_113705_AA0359620  TRIAE_CS42_2BL_TGACv1_130399_AA0410390  TRIAE_CS42_6AS_TGACv1_486422_AA1561090 | F: TGAGCGCCATCAATATCATC  R: TATGCATACGCCACCGAATA  F: CCCAGGGTGCTCATGATACT  R; AATCTGGCATTTGTTGCGTA  F: CCAGAGAACCCTTGAAGCAG  R: GCACCTCAATTGCCAAATCT  F: GACATCCACACCACGAAATG  R: GAAGGTGCTCCAGATGAAGG |
